# A Fovea for Pain at the Fingertips

**DOI:** 10.1016/j.cub.2013.02.008

**Published:** 2013-03-18

**Authors:** Flavia Mancini, Chiara F. Sambo, Juan D. Ramirez, David L.H. Bennett, Patrick Haggard, Gian Domenico Iannetti

**Affiliations:** 1 Department of Neuroscience, Physiology and Pharmacology, University College London, London WC1E 6BT, UK; 2 Institute of Cognitive Neuroscience, University College London, London WC1N 3AR, UK; 3 Wolfson CARD, King’s College London, London SE1 1UL, UK; 4 Nuffield Department of Clinical Neurosciences, University of Oxford, Oxford OX3 9DU, UK

## Abstract

The spatial resolution of sensory systems is not homogeneous across their receptive surfaces. For example, tactile acuity is greatest on the fingertips, reflecting the high innervation density and small mechanoreceptive fields in this area [1, 2]. In contrast, pain is considered to lack any equivalent to the tactile fovea on the fingertips, where the density of nociceptive fibers is remarkably low [3]. Here, by combining psychophysics with histology, we show that this established notion is incorrect. By delivering small-diameter nociceptive-specific laser pulses to human volunteers, we discovered that (1) the spatial acuity for pain is higher on the fingertips than on proximal skin regions such as the hand dorsum, and (2) this distal-proximal gradient for pain is comparable to that for touch. In contrast, skin biopsies in the same participants showed that the intraepidermal nerve fiber density is lower in the fingertips than in the hand dorsum. The increased spatial acuity for pain on the fingertips therefore cannot be explained simply by peripheral innervation density. This finding is, however, consistent with the existence of fine-grained maps of nociceptive input to individual digits in the human primary somatosensory cortex [4].

**Video Abstract:**

## Highlights

► The spatial acuity for pain is higher on the fingertips than on the hand dorsum ► The density of nociceptive fibers is lower on the fingertips than on the dorsum ► The fovea at the fingertips is not explained by nociceptive innervation density

## Results

### Psychophysical Evidence of High Spatial Resolution for Pain on the Fingertips

**Figure 1:**
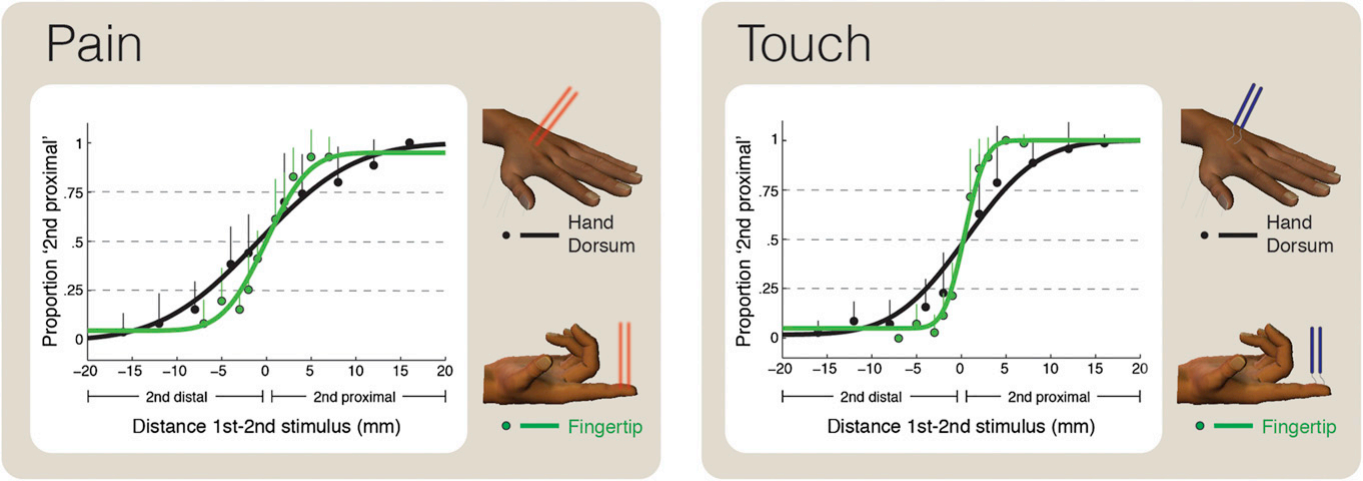
Group Psychophysical Results Spatial resolution for pain (left panel) and touch (right panel) on the fingertip (green) and hand dorsum (black). Participants were required to discriminate the locations of two successive stimuli, aligned along the proximal-distal axis of the targeted body part. The x axis shows the spatial separation between the two stimuli (negative values indicate that the second stimulus was distal to the first stimulus). The y axis shows the proportion of trials in which the second stimulus was perceived as more proximal than the first. Individual data were fitted by cumulative Gaussian functions. Data in the figure show the average (+ SD) of ten participants. The steeper curves for the discriminations on the fingertip show that spatial resolution is higher on the fingertips than on the hand dorsum for both pain and touch. See also Movie S1.

In a psychophysical experiment, we compared the spatial resolution for pain and touch on the fingertips and on the hand dorsum. We measured the ability to discriminate the skin locations of two successive stimuli aligned along the proximal-distal axis of the targeted body part (Figure 1). To ensure that painful stimuli did not activate skin mechanoreceptors, we used radiant heat laser pulses that selectively activate Aδ nociceptive afferents [5], eliciting a purely painful pinprick sensation [6] (Experimental Procedures; see also Movie S1 available online for a video of the laser stimulation). We delivered innocuous mechanical stimuli using von Frey hairs to activate Aβ mechanoreceptive afferents. We then fitted the proportion of trials in which the second stimulus was perceived as more proximal than the first as a function of the spatial separation between them, using a cumulative Gaussian function (Figure 1; Experimental Procedures). The mean of the fitted Gaussian estimates the point of subjective equality (PSE). PSEs were near zero for all four conditions (all p > 0.15), showing that there was no directional bias in location judgments. The difference in spatial separation between the 0.25 and 0.75 points defines the just-noticeable difference (JND), a measure of spatial discrimination ability. A high JND corresponds to poor spatial resolution.

**Figure 2:**
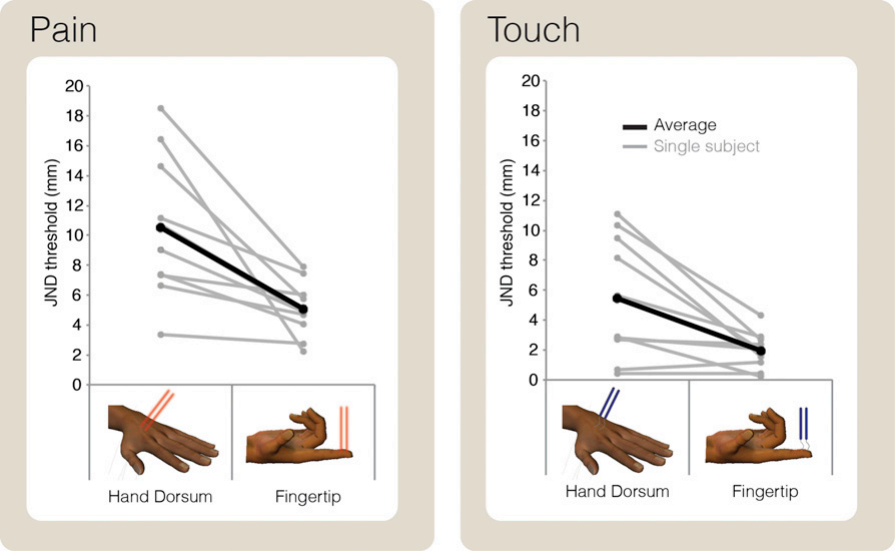
Individual Psychophysical Results Spatial discrimination thresholds (just noticeable difference, JND) for each individual subject, represented as a function of the skin region (hand dorsum or fingertip) and stimulus modality (pain or touch). Note that spatial discrimination is better on the fingertip than on the hand dorsum, for both pain and touch. See also Movie S1.

We observed the predicted proximal-distal gradient in tactile localization, with better spatial resolution on the fingertips than on the hand dorsum (paired t test: t_9_ = −3.22, p = 0.010; Figure 1; [1, 2, 7]). Surprisingly, we observed a similar gradient in pain localization, again finding better spatial resolution on the fingertips than on the hand dorsum, in all subjects (paired t test: t_9_ = −3.86, p = 0.004; Figure 1; individual data are shown in Figure 2). A two-way repeated-measures ANOVA, with skin region (fingertip or dorsum) and modality (pain or touch) as experimental factors, confirmed a main effect of skin region (F_1,9_ = 20.98, p < 0.001).

There was also a main effect of modality (F_1,9_ = 18.07, p = 0.002): JNDs were overall higher for pain than for touch, indicating lower spatial sensitivity for nociceptive than for mechanical stimulation. The interaction between skin region and modality was far from significant (F_1,9_ = 1.55, p = 0.25), suggesting that the proximal-distal gradient in sensitivity is similar in the two modalities.

In a control experiment performed using only laser stimuli, we ruled out the possibility that the proximal-distal gradient in spatial resolution for pain in the main experiment was due to transfer of learning about the spatial locations of tactile stimuli in preceding blocks (see Figure S1).

### Histological Evidence of Low Nociceptive Innervation Density on the Fingertips

**Figure 3:**
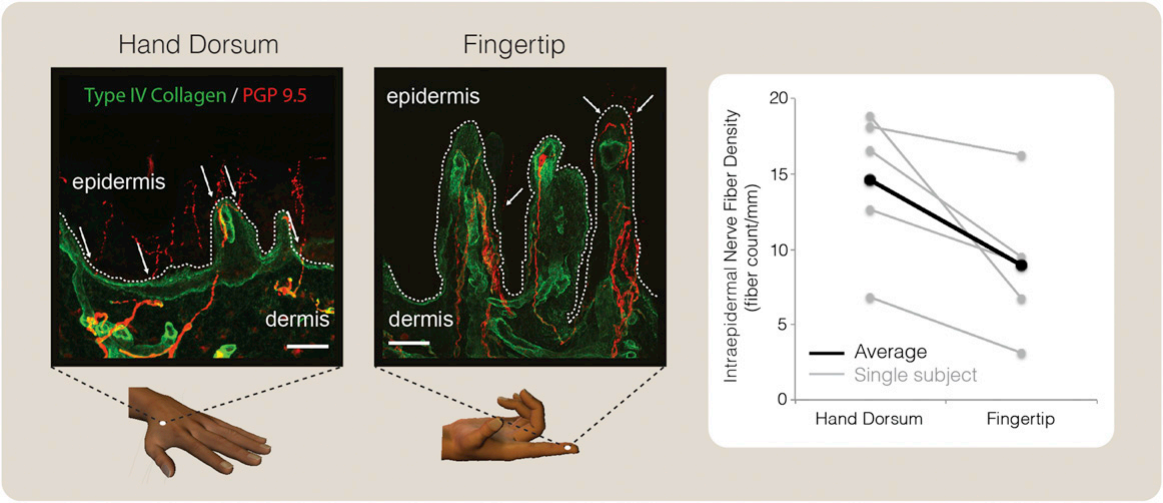
Skin Biopsies Left: confocal images of skin biopsies taken from the dorsum of the hand and fingertip, demonstrating PGP 9.5-immunoreactive fibers (red) crossing the basement membrane (labeled with type IV collagen fibers, green). Arrows indicate fibers crossing into the epidermis. Scale bars represent 50 μm. Right: intraepidermal nerve fiber density (IENFD, fiber count/mm) in the dorsum of the hand and in the fingertip. Note the clear proximal-to-distal decrease in IENFD in all participants.

Five of the ten individuals who participated in the psychophysical experiment agreed to undergo biopsy examination of tissue samples taken from the hand dorsum and the tip of the index finger. Intraepidermal nerve fiber density (IENFD) was significantly lower on the fingertip than on the hand dorsum (t_4_ = 3.13, p = 0.035; Figure 3). This within-subject comparison confirms previous between-subject reports showing that innervation density of nociceptive fibers is progressively lower when moving from proximal to distal body territories [8], including the human hand [3].

Therefore, peripheral innervation density cannot readily explain the maximal spatial resolution for pain on the fingertips.

## Discussion

We show that the spatial resolution for pain is higher on the fingertips than on proximal skin areas. This proximal-distal gradient is comparable to that for touch (Figures 1 and 2). Skin biopsies performed on the same participants showed that the innervation density of nociceptive fibers is lower on the fingertips than on the hand dorsum (Figure 3), in agreement with previous reports [3]. Therefore, the increased spatial resolution for pain on the fingertips cannot be explained simply by innervation density.

In contrast to touch, pain is commonly considered to lack a region of high spatial resolution. This notion mostly relies on anatomical evidence of low density of intraepidermal nerve fibers in distal body territories [8–10], as well as on the corresponding psychophysical evidence of progressively higher perceptive thresholds for nociceptive stimuli delivered to more distal body territories [11]. However, psychophysical investigations of painful sensations elicited by selective stimulation of nociceptive afferents on the fingertips are lacking. Thus, the hypothesis that pain lacks a foveal area of highest spatial resolution has not been tested, and it is challenged by our current results (Figures 1 and 2).

In one study, Weissman-Fogel et al. [12] investigated localization of contact-heat stimuli and found a proximal-distal gradient along the upper back, leg, and foot, but they did not explore the fingertip. Moreover, they used large contact-heat stimuli, which unavoidably involve a contribution of mechanical tactile afferents in coding spatial location. Studies that explicitly compared the spatial resolution for touch and pain using nociceptive-selective stimulation investigated only low-acuity skin regions (e.g., the hand dorsum and the forearm [7, 13–15]). In particular, two-point discrimination on the forearm is worse for pain than for touch [14], whereas single-point localization on the hand dorsum can be comparable for pain and touch [7, 15]. (Note that different physiological processes underlie the discrimination of consecutive versus simultaneous presentation of two stimuli. The higher acuity observed when presenting consecutive as opposed to simultaneous stimuli is explained by the extra information provided by the overlap of more than one receptive field when only one stimulus at a time is applied [16].) Delivering nociceptive-selective stimuli to small skin regions, such as the fingertips, is difficult because (1) the glabrous skin is not transparent to laser pulses of long wavelength (e.g., 10.6 μm of CO_2_ lasers), and (2) standard nociceptive-selective laser stimulation uses relatively large spot sizes (>4 mm [7]). Here, we overcame these issues by using a neodymium:yttrium-aluminum-perovskite (Nd:YAP) laser with a wavelength that allows selective activation of nociceptive afferents even in the glabrous skin [5] and by telescoping the laser beam down to a spot size of 1.3 mm.

The perceptual evidence for a pain fovea in the fingertips (Figures 1 and 2) is surprising, given that IENFD is relatively low in this area (Figure 3). Indeed, the classical psychophysical picture of fine spatial resolution for touch on the fingertips and for vision in the fovea is generally explained in terms of peripheral innervation density and receptive field size [16]. However, our evidence for a pain fovea cannot be easily explained in the same way. First, using a within-subjects design, we confirmed that the density of intraepidermal innervation decreases moving from the hand dorsum to the fingertips [3]. A similar proximal-to-distal decrease of IENFD has also been reported in other body parts [8–10], in obvious contrast to the proximal-to-distal increase in the density of large myelinated fibers that transmit tactile input [17]. Second, little is known about the size of nociceptive receptive fields on the fingertips, although a few intraneural microstimulation studies of Aδ fibers have reported a uniform size of the projected fields of sensation in proximal and distal territories of the hand [18, 19].

The density of peripheral receptors innervating a given portion of the receptive surface influences the size of the area of the primary sensory cortex that is devoted to processing the sensory input. Thus, large areas of the primary somatosensory cortex (SI) represent the tactile input from the digits [20]. This cortical magnification underlies the fine spatial resolution of the fingertips [17], although it remains unclear whether the cortical magnification merely follows from corresponding peripheral innervation levels or involves some additional central mechanisms [21]. Functional neuroimaging studies in humans have confirmed that tactile acuity is related to the extent of cortical magnification [22]. We recently demonstrated that human SI contains fine-grained maps reflecting nociceptive-selective input to individual digits [4]. The fine somatotopy of nociceptive input in the SI is the likely neuronal substrate of a fovea for pain on the fingertips.

What mechanisms might enhance the spatial resolution of the nociceptive system beyond the level suggested by peripheral innervation density? One parsimonious explanation involves spinal or supraspinal mechanisms governing local interactions within the nociceptive system. These might include surround inhibition [16] or other forms of population coding [23]. However, at least in the tactile and visual systems, such local interaction mechanisms follow innervation density [16]. An alternative explanation suggests that pain acuity may depend not only on nociceptive neurons but also on multimodal neural populations at spinal [24, 25] or cortical [26] level. This possibility is supported by the high alignment of the fine-grained maps of tactile and nociceptive input to individual digits in the SI [4]. Understanding these mechanisms is important for a correct description of the neurophysiological mechanisms that underlie pain.

## Experimental Procedures

### Participants

Ten healthy participants (six males and four females, mean ± SD age 29.4 ± 4.1 years) took part in the main psychophysical experiment. Five of them also underwent histological assessment of innervation density on the hand dorsum and fingertips. Written informed consent was obtained from all participants. The study was conducted in accordance with the principles of the Declaration of Helsinki. All experimental procedures were approved by the local ethics committees.

### Nociceptive Laser Stimuli

Radiant-heat stimuli were generated by an infrared neodymium:yttrium-aluminum-perovskite (Nd:YAP) laser (Electronic Engineering, Calenzano, Italy) with a wavelength of 1.34 μm. At this short wavelength, the skin is highly transparent to the laser beam, and hence passive heat propagation is not needed to reach the depth at which nociceptive terminals are located [5]. The laser pulse (4 ms duration) was transmitted via an optic fiber and focused by lenses to reach a spot diameter of 1.3 mm. A He-Ne laser guide was used to direct the stimulation to the desired location on the skin. The laser energies (0.3–0.45 J) were adjusted in each subject to (1) elicit a clear pinprick pain sensation, reflecting Aδ fiber activation [6]; (2) achieve a pain intensity rating of 3 out of 10 (where 0 is no pain and 10 is the worst pain imaginable); and (3) match the intensity of the elicited sensation on the two stimulated territories. After achieving a pain rating of 3 out of 10, we made sure that the intensity of the sensation was stable by asking the subjects at the end of each block. The skin temperature of the area stimulated was monitored every 10 min with an infrared thermometer and kept at ∼32°C ± 1°C. Participants were instructed to keep their hands still. It is unlikely that the laser stimuli at the fluence we used (0.01 J/cm^2^) elicited any motor response. Indeed, a withdrawal response can only be recorded when stimuli are delivered at much higher energies (e.g., 350 J/cm^2^; [27]).

### Innocuous Mechanical Stimuli

A von Frey hair (diameter 0.49 mm, length 40 mm, weight 8 g) was used to deliver innocuous mechanical stimuli. At this stimulus intensity, all stimuli elicited a clear, nonpainful percept.

### Psychophysical Procedure

A 2 × 2 factorial design with modality (pain and touch) and skin region (fingertip and dorsum) was used in the psychophysical experiment. An experimenter who was blinded to the experimental hypotheses tested five participants. Furthermore, to control for response bias, half of the participants were required to judge whether the first stimulus was delivered distally to the second stimulus; the other half judged instead whether the second stimulus was delivered proximally to the first stimulus. The interstimulus interval between the two stimuli was ∼2 s. For each subject, stimuli were delivered in two separate sessions. In each session, stimuli were delivered either to the volar surface of the fingertip or to the hand dorsum. The order of sessions was counterbalanced across participants. We alternated the stimulation of the tips of the index and middle fingers to avoid nociceptor fatigue or sensitization, as well as increases of baseline temperature. Similarly, we stimulated different locations on the center of the hand dorsum, within a 4 × 3 cm region. For both districts, at least 2 min elapsed between successive stimulations of the same location. Within each session, laser and mechanical stimuli were administered in alternating blocks. The number of blocks per session was 14 (seven blocks for each sensory modality), and the order of blocks was also counterbalanced across participants. In each block, we delivered ten pairs of spatially separated stimuli. The spatial separations were 7, 5, 3, 2, and 1 mm on the fingertip and 16, 12, 8, 4, and 2 mm on the hand dorsum. The total number of stimulus pairs per participant was 280.

To obtain a psychometric function, we fitted the data with cumulative Gaussian functions that were free to vary in position along the x axis and slope. We obtained the fit using a bootstrap procedure and maximum-likelihood estimation [28].

### Skin Biopsies

Two 3 mm punch skin biopsies were taken from each participant to assess IENFD. Biopsies were taken from the lateral aspect of the index finger and the dorsum of the hand (Figure 3). The tissue obtained was washed with 0.1 M Sorenson’s phosphate buffer, fixed in 2% periodate-lysine-paraformaldehyde overnight, and cryoprotected overnight in 30% sucrose/PBS solution before being embedded in OCT. We immunostained 50 μm cryosections using antibodies directed against the pan-neuronal protein gene product 9.5 (rabbit anti-PGP 9.5 Ab, 1:2,000; Ultraclone) and the basal membrane marker collagen type IV (goat anti-collagen type IV Ab, 1:400; Millipore), followed by Cy3 goat anti-rabbit secondary antibodies (1:500; Jackson ImmunoResearch), and Alexa 488 donkey anti-goat (1:1,000; Alexa Fluor, Invitrogen/Life Technologies). All sensory afferents are PGP 9.5 immunoreactive. Therefore, the quantification of PGP 9.5-positive free nerve endings entering the epidermis will represent both C and Aδ fiber populations. Images were taken using a laser scanning confocal microscope (Zeiss LSM 710 with 10× and 20× Plan-Apochromat objectives; Carl Zeiss MicroImaging) at 2 μm intervals. Final images are displayed as an overlay of single maximum projections. PGP 9.5-positive nerve fibers crossing the basal membrane were counted according to previously published guidelines [29] by one investigator (J.D.R.). Counts were given as number of fibers per millimeter length of epidermis.

## Supplemental Information

Document S1. Figure S1, Control Experiment Conducted Using Laser Stimulation Only

Movie S1. Example of the Nd:YAP Laser Stimulation on Thermal Paper Two laser pulses are successively delivered with a 2 s time interval. The laser is moved manually and triggered automatically via computer.
