# Supplementary figures and images for: A Fovea for Pain at the Fingertips
Source: Curr Biol. 2013 Mar 18;23(6):496–500. doi: 10.1016/j.cub.2013.02.008 (PMC3778751; doi:10.1016/j.cub.2013.02.008)

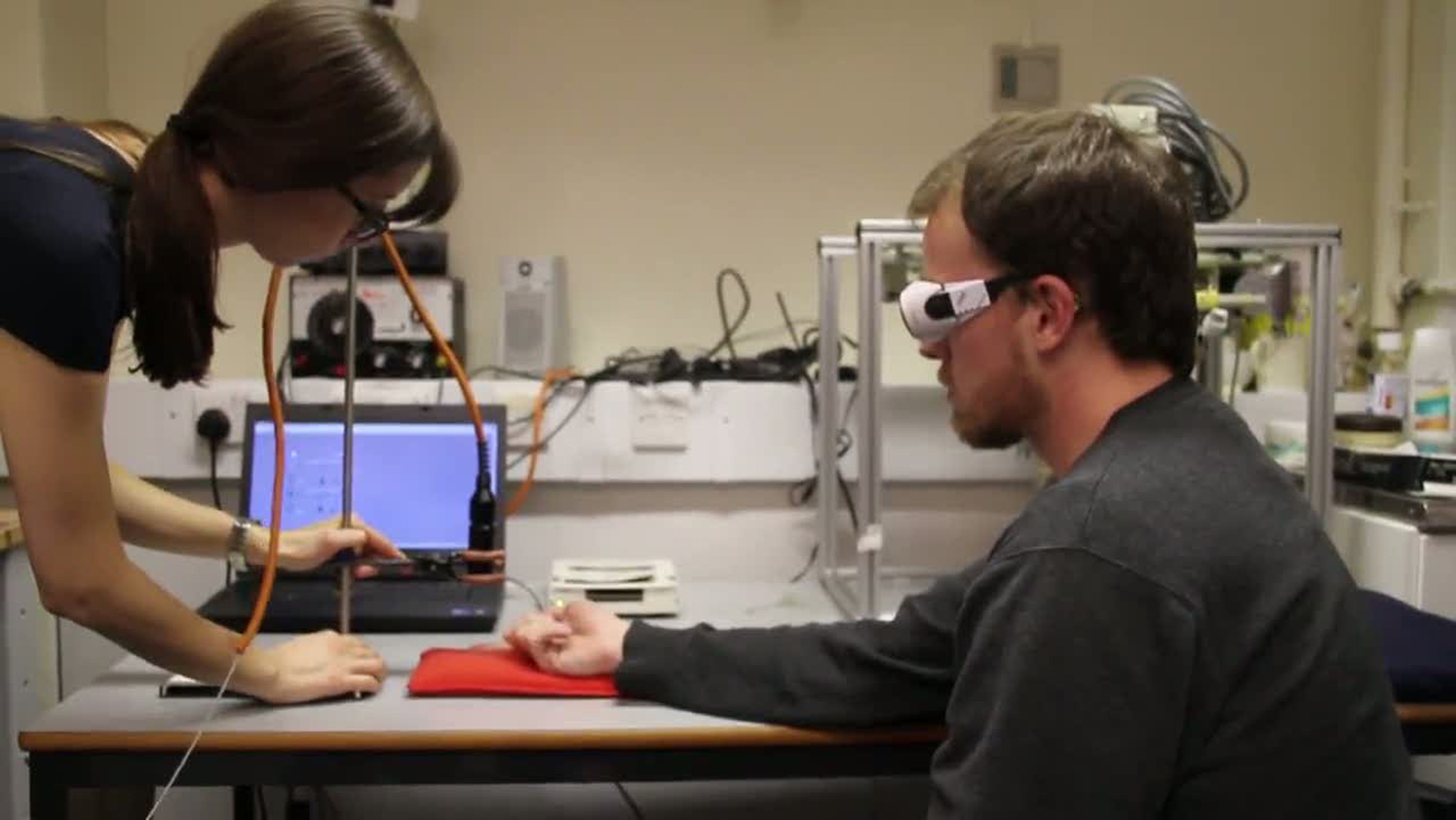

Supplement: Supplementary file 1 [file mmc3.jpg]
