# Supplementary material for: A Fovea for Pain at the Fingertips
Source: Curr Biol. 2013 Mar 18;23(6):496–500. doi: 10.1016/j.cub.2013.02.008 (PMC3778751; doi:10.1016/j.cub.2013.02.008)
Supplement: Document S1. Figure S1, Control Experiment Conducted Using Laser Stimulation Only [file mmc1.pdf]

Current Biology, Volume *23*

Supplemental Information

A Fovea for Pain at the Fingertips

Flavia Mancini, Chiara F. Sambo, Juan D. Ramirez, David L.H. Bennett, Patrick Haggard, and Gian Domenico Iannetti

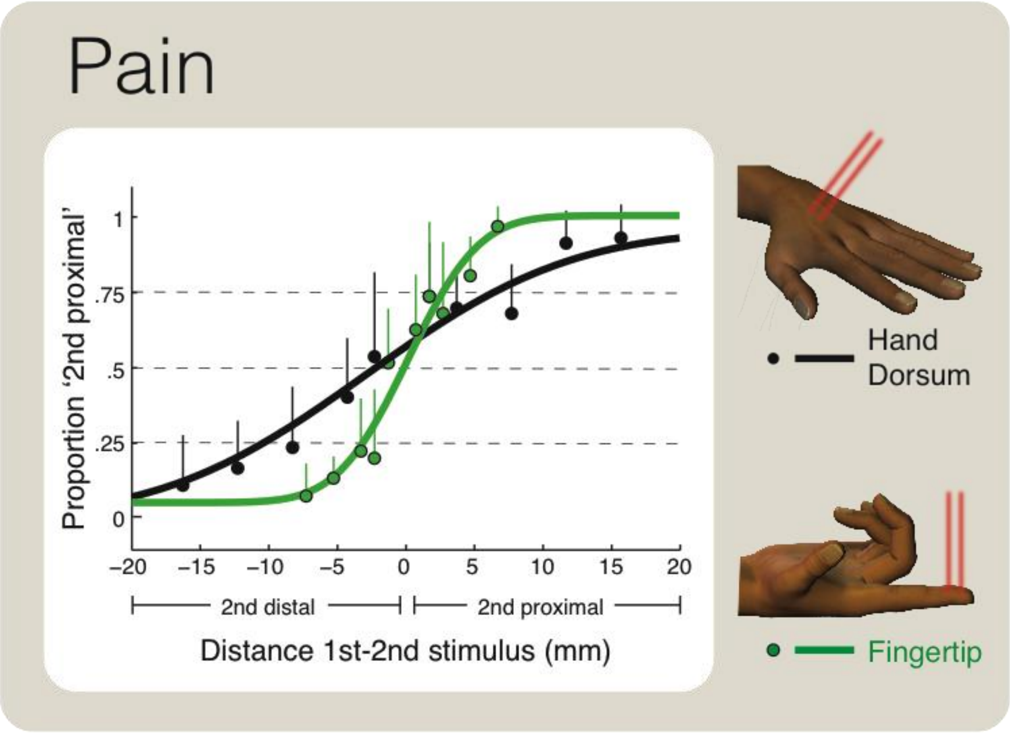

Figure S1. Control Experiment Conducted Using Laser Stimulation Only

Spatial resolution for pain on the fingertip (green) and the hand dorsum (black).

Eight additional participants (mean age = 23.5 years, SD ± 3, 5 females) were tested in a control experiment, which included only nociceptive but not tactile localization. The aim of this control experiment was to test whether the proximal-distal gradient in spatial resolution for pain observed in the main experiment was due to the learning of the spatial intervals used in the preceding tactile blocks. All participants were naïve to laser stimulation and to the experimental task. They were required to discriminate the locations of two successive stimuli, aligned along the proximal-distal axis of the targeted body part. *X-axis*: spatial separation between the two stimuli (negative values indicate that the second stimulus was distal to the first stimulus). *Y-axis*: proportion of trials in which the second stimulus was perceived as more proximal than the first. Individual data were fitted by cumulative Gaussian functions. Data in the figure show the average (+ SD) of 8 participants. We replicated our previous finding of better spatial resolution on the fingertips (mean JND = 5.34, SD ± 2.25 mm) than on the hand dorsum (mean JND = 12.43, SD ± 5.14 mm; paired t-test: $t_7 = -4.99$, $p = 0.002$).
